# Red Nucleus Excitatory Neurons Initiate Directional Motor Movement in Mice

**DOI:** 10.3390/biomedicines13081943

**Published:** 2025-08-08

**Authors:** Chenzhao He, Guibo Qi, Xin He, Wenwei Shao, Chao Ma, Zhangfan Wang, Haochuan Wang, Yuntong Tan, Li Yu, Yongsheng Xie, Song Qin, Liang Chen

**Affiliations:** 1 Department of Anatomy and Histology Embryology, School of Basic Medical Sciences, Fudan University, Shanghai 200032, China; 22211010039@m.fudan.edu.cn (C.H.); 20211010033@fudan.edu.cn (G.Q.); hx519374741@163.com (X.H.); 22301050323@m.fudan.edu.cn (W.S.); jinmachao99@163.com (C.M.); 24211010039@m.fudan.edu.cn (Z.W.); whc990521@126.com (H.W.); tyt_108@i.smu.edu.cn (Y.T.); 19518558166@163.com (L.Y.); 2 Neurosurgical Institute, Fudan University, Shanghai 200040, China; 3 State Key Laboratory of Medical Neurobiology and MOE Frontiers Center for Brain Science, Institutes of Brain Science, Fudan University, Shanghai 200032, China

**Keywords:** red nucleus, excitatory neurons, lateralized movement, anterograde

## Abstract

**Background**: The red nucleus (RN) is a phylogenetically conserved structure within the midbrain that is traditionally associated with general motor coordination; however, its specific role in controlling directional movement remains poorly understood. **Methods**: This study systematically investigates the function and mechanism of RN neurons in directional movement by combining stereotactic brain injections, fiber photometry recordings, multi-unit in vivo electrophysiological recordings, optogenetic manipulation, and anterograde trans-synaptic tracing. **Results**: We analyzed mice performing standardized T-maze turning tasks and revealed that anatomically distinct RN neuronal ensembles exhibit direction-selective activity patterns. These neurons demonstrate preferential activation during ipsilateral turning movements, with activity onset consistently occurring after movement initiation. We establish a causal relationship between RN neuronal activity and directional motor control: selective activation of RN glutamatergic neurons facilitates ipsilateral turning, whereas temporally precise inhibition significantly impairs the execution of these movements. Anterograde trans-synaptic tracing using H129 reveals that RN neurons selectively project to spinal interneuron populations responsible for ipsilateral flexion and coordinated limb movements. **Conclusions**: These findings offer a framework for understanding asymmetric motor control in the brain. This work redefines the RN as a specialized hub within midbrain networks that mediate lateralized movements and offers new avenues for neuromodulatory treatments for neurodegenerative and post-injury motor disorders.

## 1. Introduction

The red nucleus (RN) is an ancient nucleus that, as a phylogenetically conserved structure located within the ventral midbrain, serves as a critical node in the extrapyramidal motor system [1,2,3,4]. Disruption of RN function has been linked to a range of movement disorders, including essential tremor (ET), Parkinson’s disease (PD), and impaired motor recovery following corticospinal tract injury [5,6,7,8,9,10,11,12,13]. Although traditionally viewed as a relay within basic motor circuitry, contemporary research has revealed its precise regulatory role in coordinating complex multi-joint movements, such as grasping and reaching behaviors [14,15,16,17]. In quadrupedal animals, the RN interacts with the corticospinal tract via the rubrospinal pathway to regulate postural stability and locomotor coordination, particularly during precision movements that require adaptive limb control and environmental navigation [3,18,19,20,21].

The generation of coordinated, oriented motor behaviors is fundamental to animal survival. Turning behavior, a fundamental yet complex form of asymmetric movement, requires precise bilateral coordination across multiple motor structures. Previous studies have emphasized the involvement of both basal ganglia circuits and specific brainstem nuclei in generating and modulating such lateralized movements [22,23,24,25]. For example, genetically defined excitatory V2a reticular neurons in the brainstem have been shown to orchestrate orientation-related motor actions. Notably, our recent findings demonstrate that during an auditory decision-making task, RN neurons exhibit robust and selective activation when mice orient toward the contralateral reward port [26], suggesting a specialized role for the RN in mediating directionally biased movements. In summary, although the role of the red nucleus in basic motor coordination has been recognized, the specific contribution of the RN to directional control remains incompletely understood.

Although the RN is predominantly composed of neurons expressing CaMKIIα and VGluT2 [19,27,28,29], the functional heterogeneity within this seemingly homogeneous population and its precise role in lateralized motor control remain largely unclear. To address these fundamental questions, we employed an integrated experimental approach combining fiber photometry and multi-unit in vivo electrophysiological recordings to monitor the dynamic activity of RN neurons during precisely controlled T-maze turning behaviors in mice. Furthermore, we applied cell-type-specific optogenetic manipulations to establish causal links between RN neuronal activity and directional movement parameters. In addition, H129 trans-synaptic tracing was utilized to systematically map the downstream effector pathways through which the RN influences the spinal motor circuitry involved in turning behavior.

Our findings fundamentally reconceptualize the role of the RN, establishing it as a specialized hub for the integration and execution of lateralized motor commands. This work provides unprecedented mechanistic insights into the functional organization of midbrain motor networks and offers a neurobiological framework that may inform the development of novel therapeutic strategies for movement disorders characterized by asymmetric motor dysfunction.

## 2. Materials and Methods

### 2.1. Animals

Wild type (WT) mice on the C57BL/6 background were obtained from Shanghai Jihui Company (Shanghai, China). VGluT2-Cre (RRID: IMSR_JAX:028863) mice were purchased from The Jackson Laboratory (Bar Harbor, ME, USA) and bred to expand their numbers. The mice were housed in an environment with a 12-h light and 12-h dark cycle, at a constant temperature and humidity, and were given unlimited access to food and water. To avoid potential interference from hormonal fluctuations associated with the estrous cycle in female mice, we used male mice aged 8–12 weeks in this behavioral experiment.

### 2.2. Brain Preparation and Immunocytochemistry

As previously described, mice were anesthetized and subjected to transcranial perfusion with phosphate-buffered saline (PBS), followed by a 4% paraformaldehyde (PFA) solution. The whole brain was then dissected and post-fixed with 4% PFA for 24–48 h at 4 °C. After fixation, the brain tissue was cryoprotected in a 30% sucrose solution and sectioned into 40 µm-thick slices using a cryostat (Leica Microsystems, Wetzlar, Germany).

For immunocytochemistry, brain sections were washed with PBS and permeabilized with 0.3% Triton X-100 for 30 min. After blocking with 5% bovine serum albumin (BSA) for 1 h, the sections were incubated with primary antibodies overnight at 4 °C. Subsequently, corresponding secondary antibodies (Alexa Fluor 488-, 555-, or 647-conjugated antibodies) were applied for 2 h at room temperature. Finally, nuclei were counterstained with Hoechst 33,258 (1 µg/mL).

### 2.3. Stereotactic Brain Injections

Mice were anesthetized with isoflurane and secured in a stereotaxic frame. Following skull exposure, a craniotomy was performed (3.6 mm posterior to the bregma, 0.6 mm lateral to the midline, 3.5 mm ventral to the skull surface). Using a micro-syringe pump (Yuyan Technology, Shanghai, China), viruses were injected at a rate of 1 nl/s. After the viral injection, an optical fiber (outer diameter: 1.25 mm, numerical aperture: 0.37) was implanted 100 μm above the injection site. The fiber was initially secured using light-cured adhesive or super glue, followed by permanent fixation with dental cement mixed with carbon powder. Post-surgery, mice were individually housed in recovery cages and monitored daily for one week to ensure proper recovery.

### 2.4. Fiber Photometry Recording

Before recording fiber photometry, mice were acclimatized to the T-maze for at least 5 days. The optical fiber was photobleached for 2 h before connecting to the fiber photometry system, which was used for real-time stimulation and GCaMP signal recording. Video signals and calcium activity were recorded simultaneously. Time points were automatically annotated via the software when mice turned either ipsilaterally or contralaterally relative to the implanted side. The recorded signals were analyzed using custom MATLAB (R2023a; MathWorks, Natick, MA, USA) scripts, with ΔF/F calculated using the following formula:

ΔF/F = (F − F0)/F0

where F represents the raw fluorescence signal and F0 denotes the baseline fluorescence.

### 2.5. Multi-Unit In Vivo Electrophysiological Recordings

The tetrode electrode consists of eight 32-channel metal microwire electrodes bonded with a 200-μm-diameter optical fiber. A 3D-printed tetrode holder is used to secure the assembled electrode to the fixture. During electrode implantation, the tetrode is slowly advanced through the meninges at a rate of 1 mm per minute until it is positioned 300 μm above the target brain region, taking care to avoid major blood vessels on the meningeal surface. The electrode–brain tissue interface is uniformly coated with ophthalmic ointment to protect the exposed electrodes. Subsequently, high-viscosity dental cement is applied to fill the gap between the holder and the skull, followed by complete coverage of the skull surface with dental cement. After a two-week postoperative recovery period, neuronal spike signals and local field potentials (LFPs) are recorded using the OpenEphys system (https://open-ephys.org, accessed date: 1 December 2024). Single-unit spike signals are processed through the WaveClus pipeline, which includes spike detection, feature extraction, and clustering, to identify well-isolated single neurons.

### 2.6. Optogenetic Manipulation

To manipulate excitatory neurons, we employed ChR2 and stGtACR2 for optogenetic modulation in experimental mice. Optogenetic manipulation was performed using recombinant adeno-associated viruses (rAAVs) for targeted neuronal modulation. For optogenetic activation, we used rAAV-EF1α-DIO-hChR2(H134R)-EYFP-WPRE-hGH (BrainVTA Co., Ltd., Wuhan, China; titer ≥ 5 × 10^12^ vg/mL) [30] and rAAV-CaMKIIα-hChR2(H134R)-EYFP-WPRE-hGH (BrainVTA Co., Ltd.; titer ≥ 5 × 10^12^ vg/mL) [31], while optogenetic inhibition was achieved with rAAV-hSyn-DIO-stGtACR2-EGFP (Brain Case Co., Ltd., Shenzhen, China; titer ≥ 5 × 10^12^ vg/mL), with a standardized injection volume of 300 nl. Control mice expressed GFP only. Opto-stimulation of RN neurons uses two procedures. The short one (10 s duration, 5–8 mW at fiber tip, 5 ms pulse, 20 Hz, 6 trains with 2 s interval) was adopted to conduct the DeepLabCut-based behavioral analysis (https://github.com/DeepLabCut/DeepLabCut, accessed date: 25 March 2025). The long one (300 s duration, 5–8 mW at fiber tip, 5 ms pulse, 20 Hz, 6 trains with 2 s interval) was delivered to compare the number of revolutions. The frequency varied among 1 Hz, 5 Hz, 20 Hz, and 40 Hz. And the numbers of 360° rotations were manually counted.

### 2.7. DeepLabCut-Based Behavioral Analysis

Experimental videos were imported into DeepLabCut (Python environment), where the nose tip, left/right ears, and tail base of each mouse were manually labeled frame-by-frame to train the automated tracking model. Real-time angular velocity was calculated from frame-to-frame rotational changes in the nose-to-tail base vector. The mean angular velocity during active rotation periods (excluding immobile phases) was defined as the average angular velocity. Head–trunk angular displacement was computed as the intersecting angle between the nose-to-bisection point (midpoint between ears) line and the bisection-to-tail base line.

### 2.8. Electromyography (EMG)

Following ketamine anesthesia (110 mg/kg, i.p.), mice were secured in a supine position on the recording platform. Bipolar needle electrodes were inserted into the belly of the biceps brachii muscles in both forelimbs. EMG signals were acquired synchronously with short-term optogenetic stimulation. The maximum amplitude within a 5 s post-stimulation window was analyzed using custom MATLAB (R2023a) scripts (MathWorks). Each mouse received at least 5 valid stimulus repetitions.

### 2.9. Anterograde Trans-Synaptic Tracing

H129 is a clinical isolate of the HSV-1 virus, characterized by its specific anterograde trans-synaptic property. A volume of 200 nl of the H129-G4 (Titer ≥ 2 × 10^9^ pfu/mL) viral suspension was unilaterally injected into the right RN of wild-type mice [32]. Tissue samples were collected and subjected to imaging at 36 and 72 h post-infection.

### 2.10. Statistical Analyses

The Wilcoxon signed-rank test was performed for all two-group comparisons. For comparisons among three or more groups, one-way analysis of variance (ANOVA) was performed. Post hoc analyses were performed using Tukey’s or Dunn’s tests for multiple comparisons. All statistical analyses were performed using GraphPad Prism software (version 8.2, GraphPad Software, La Jolla, CA, USA). The data were presented as means ± standard errors of the means.

## 3. Results

### 3.1. The RN Is More Active During Ipsilateral Turning

**Figure 1:**
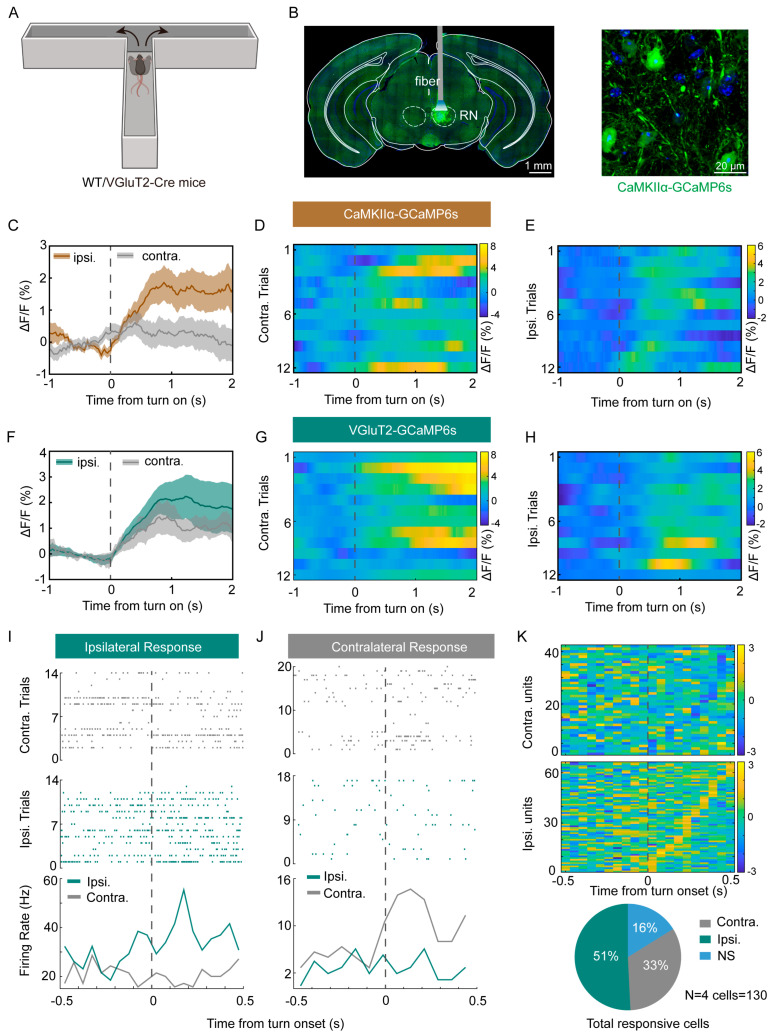
The RN is more active during the ipsilateral turning. (**A**) Experimental setup and schematic of the T-maze. (**B**) Left panel: representative image of virus expression in the RN; the green fluorescent protein (GFP)-expressing viral infection was precisely localized within the red nucleus (RN) (scale bar: 1 mm). Right panel: representative image of CaMKII+ neurons expressing GCaMP6s in the RN (scale bar: 20 μm). (**C**,**F**) Average calcium signals in response to ipsilateral turns and contralateral turns in RN CaMKII+ neurons (**C**) and VGluT2+ neurons (**F**). The average fluorescence during turns was significantly increased (unpaired *t* test, *p* < 0.0001, *n* = 6 mice per group). All data are presented as means ± SEMs. (**D**,**E**) Heatmap of the RN CaMKII+ neuronal responses to contralateral trials (**D**) and ipsilateral trials (**E**) for one example mouse. (**G**,**H**) Heatmap of the RN VGluT2+ neuronal responses to contralateral trials (**G**) and ipsilateral trials (**H**) for one example mouse. (**I**,**J**) One example RN neuron with an ipsilateral preference (**I**) and contralateral preference (**J**). Top panel: raster plots of neuronal activity for individual contralateral (gray) and ipsilateral (green) trials aligned to the time from turn on. Bottom panel: PSTH for ipsilateral (green) and contralateral (gray) trials. There is a significant statistical difference in firing rates between ipsilateral and contralateral trials (unpaired *t* test, *p* < 0.0001). (**K**) Neurons were classified based on their contralateral/ipsilateral response preference, calculated using a normalized modulation index (MI) defined as MI = (Ripsi − Rcontra)/(Ripsi + Rcontra), where Ripsi and Rcontra represent the mean firing rates during ipsilateral and contralateral turns, respectively. The pie chart shows the number of neurons (130 units total) with a contralateral preference (gray, 33%), ipsilateral preference (green, 51%), and no preference (blue, 16%). N = 4, cells = 130.

To investigate the involvement of the RN in locomotor asymmetry, we recorded neuronal activity in the RN during turning behaviors in a T-maze task (Figure 1A). Viral expression mapping confirmed the precise targeting of calcium indicators to the RN (Figure 1B). As calcium/calmodulin-dependent protein kinase II (CaMKII) is a well-established marker of excitatory neurons [33], we initially assessed the activity of CaMKII-expressing RN neurons during ipsilateral and contralateral turns. Our findings revealed a significantly greater elevation in calcium signals during ipsilateral turns relative to contralateral turns (Figure 1C–E). To corroborate these results, we employed vesicular glutamate transporter 2 (VGluT2) as an additional marker of excitatory neurons [34] and observed a similar pattern in VGluT2+ neurons, with higher activity during ipsilateral compared to contralateral turning (Figure 1F–H).

To further analyze the firing dynamics of individual RN neurons during turning behavior, we recorded electrophysiological activity in the same T-maze experimental paradigm and used Boris software v8.26, available online: http://www.boris.unito.it/, to mark turning time points. Raster and peri-stimulus time histogram (PSTH) analyses revealed that ipsilateral-responsive RN neurons exhibited significantly increased firing rates during ipsilateral turns, with spike activity predominantly occurring within 500 ms after turn initiation. In contrast, contralateral-responsive RN neurons showed higher firing rates specifically during contralateral turns (Figure 1I,J). Based on the differential firing rates observed during ipsilateral versus contralateral turns, RN neurons were classified into three distinct functional populations—ipsilaterally tuned neurons (51%), contralaterally tuned neurons (33%), and non-responsive neurons (16%)—suggesting that majority of RN neurons are highly active during ipsilateral turning (Figure 1K).

### 3.2. RN Excitatory Neurons Drive Ipsilateral Locomotion

**Figure 2:**
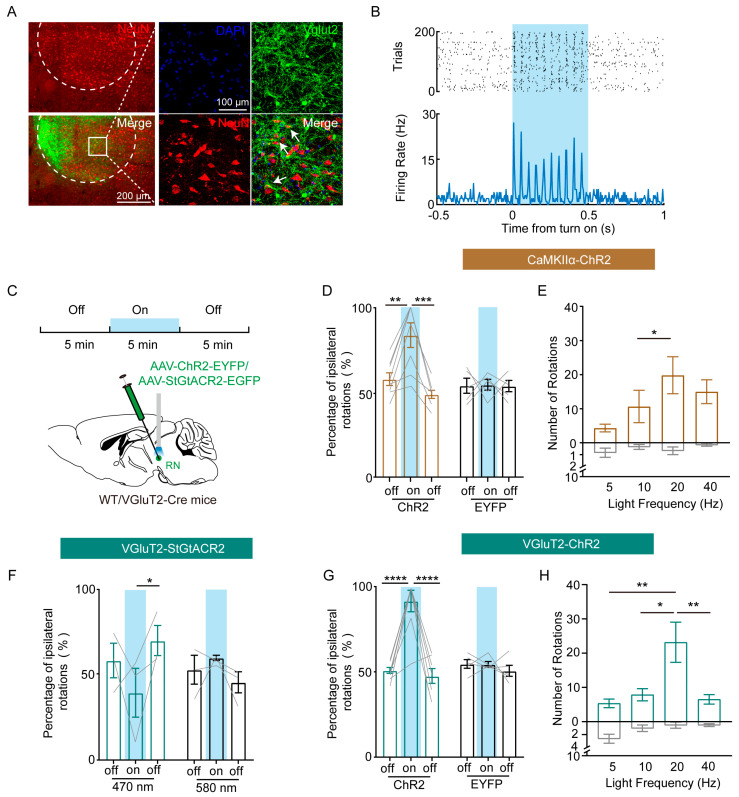
RN excitatory neurons drive ipsilateral locomotion. (**A**) Image of immunofluorescence staining showing the co-localization of VGluT2 and NeuN in RN neurons. (**B**) Stimulation with 470 nm light (500 ms) enhances the firing rate of VGluT2+ neurons expressing ChR2. (**C**) Schematic diagram of the optogenetic virus injection in the RN and timeline schematic of a single recording session. (**D**) Percentage of ipsilateral rotations (Nipsi/[Nipsi + Ncontra] × 100%) during 5 min epochs induced by the optogenetic activation of RN CaMKII+ neurons (ChR2: *n* = 8, EYFP: *n* = 5), where Nipsi and Ncontra represent the number of ipsilateral and contralateral turns, respectively. One-way ANOVA, multiple comparisons, ** *p* < 0.01 and *** *p* < 0.001. (**E**) Ipsilateral and contralateral rotation counts during the optogenetic activation of RN CaMKII+ neurons at varying stimulation frequencies (5–40 Hz; *n* = 6). One-way ANOVA, multiple comparisons, * *p* < 0.05. (**F**) Percentage of ipsilateral rotations during 5 min epochs induced by the optogenetic inhibition of RN VGluT2+ neurons (*n* = 3). One-way ANOVA, multiple comparisons, * *p* < 0.05. (**G**) Percentage of ipsilateral rotations during 5 min epochs induced by the optogenetic activation of RN VGluT2+ neurons. (ChR2: *n* = 8, EYFP: *n* = 5). One-way ANOVA, multiple comparisons, **** *p* < 0.0001 and **** *p* < 0.0001. (**H**) Ipsilateral and contralateral rotation counts during the optogenetic activation of RN VGluT2+ neurons at varying stimulation frequencies (5–40 Hz; *n* = 6). One-way ANOVA, multiple comparisons, * *p* < 0.05 and ** *p* < 0.01.

To investigate the role of RN neurons in mediating lateralized movement, we first confirmed the co-localization of VGluT2+ neurons with NeuN+ neurons within the RN via immunofluorescence staining (Figure 2A). In VGluT2-Cre mice, we targeted RN VGluT2+ neurons by injecting AAV-DIO-ChR2 into the RN and delivered 470 nm, 20 Hz blue light stimulation to selectively activate these neurons. Functional validation of ChR2-mediated activation was achieved through single-cell electrophysiological recordings, which confirmed reliable light-evoked responses (Figure 2B). To selectively regulate the activity of CaMKII+ and VGluT2+ neurons, we implemented a dual-virus strategy combining the excitatory opsin ChR2 (targeting cation channels) with the inhibitory opsin StGtACR2. Control mice received an AAV vector expressing enhanced yellow fluorescent protein (EYFP) alone (Figure 2C). Optogenetic stimulation of RN CaMKII+ neurons induced robust ipsilateral rotation (toward the stimulated hemisphere) in free-moving mice (Figure 2D,E), with some animals briefly exhibiting freezing behavior upon laser onset. These motor effects were immediately reversed upon the cessation of light stimulation. In contrast, optogenetic inhibition of RN VGluT2+ neurons suppressed ipsilateral movements (Figure 2F), while control stimulation with 589 nm light had no detectable impact on locomotor performance. Optogenetic activation of RN VGluT2+ neurons also produced rapid and robust lateralized movement, further supporting the critical role of excitatory neurons in RN-mediated lateralized motor control (Figure 2G,H).

**Figure 3:**
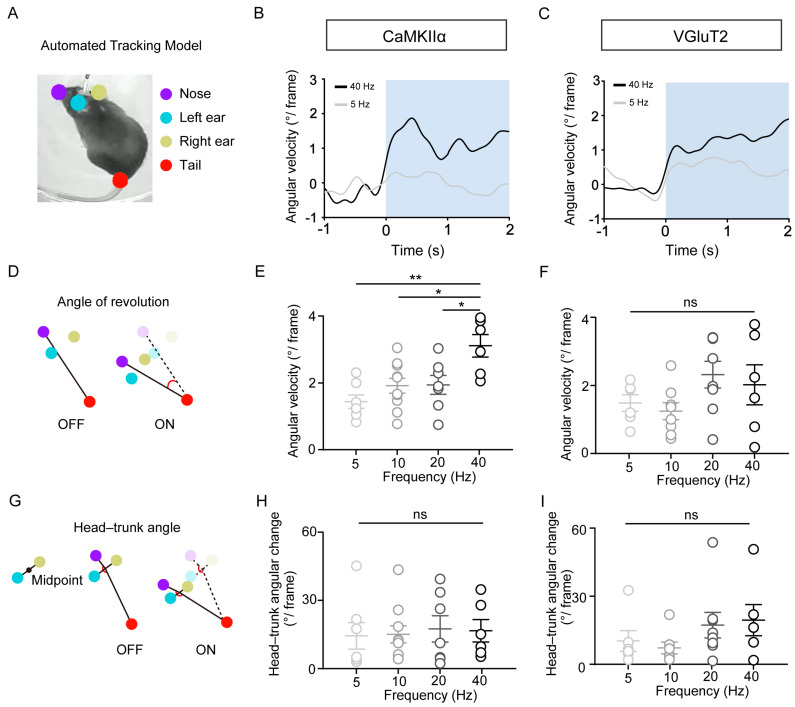
Frequency-dependent effects of optogenetic stimulation on the rotation velocity revealed by the DeepLabCut analysis. (**A**) Automatic labeling of four key body points using DeepLabCut. (**B**) Revolution behavior of mice (*n* = 4) with ChR2 expressed in CaMKII+ neurons during 5 and 40 Hz 470 nm light stimulation. (**C**) Revolution behavior of mice (*n* = 5) with ChR2 expressed in VGluT2+ neurons under the same stimulation conditions. (**D**) Schematic defining the angle of revolution. (**E**,**F**) Average angular velocity of rotation during 10 s of 470 nm light pulses at 5, 10, 20, and 40 Hz in mice with ChR2 expressed in CaMKII+ neurons (**E**) and VGluT2+ neurons (**F**). One-way ANOVA, multiple comparisons, * *p* < 0.05 and ** *p* < 0.01. All data are presented as means ± SEMs. (**G**) Schematic of the head–trunk angle measurement. (**H**,**I**) Head–trunk angle changes during 10 s of 470 nm light pulses at 5, 10, 20, and 40 Hz in mice with ChR2 expressed in CaMKII+ neurons (**H**) and VGluT2+ neurons (**I**). One-way ANOVA, multiple comparisons, ns. All data are presented as means ± SEMs.

To quantify the kinematic features of turning behavior, we utilized a DeepLabCut-based tracking system that automatically identified and labeled four anatomical landmarks, the nose tip, left ear, right ear, and tail base, enabling precise monitoring of rotational dynamics (Figure 3A). To assess whether CaMKII+ neuron activation in the RN exhibits frequency-dependent motor effects, we applied 470 nm laser stimulation (10 s duration) at 5, 10, 20, and 40 Hz. Notably, 40 Hz stimulation evoked the fastest real-time angular velocity of rotation (Figure 3B). Statistical analysis confirmed that the mean angular velocity at 40 Hz was significantly greater than at 5 Hz (*p* = 0.0013), 10 Hz (*p* = 0.0151), and 20 Hz (*p* = 0.0299) (Figure 3E). However, head–trunk angle measurements revealed no significant differences across frequencies, indicating preserved postural alignment despite changes in turning velocity (Figure 3H). For VGluT2+ neuron activation, although the 40 Hz group exhibited a trend toward a higher mean angular velocity compared to the 5 Hz group, this difference was not significant (*p* = 0.7934) (Figure 3C,F). Similarly, head–trunk angles remained unchanged across stimulation frequencies (Figure 3I). We propose that the differences in frequency responses between CaMKIIα+ and VGluT2+ neuronal populations may stem from fundamental biological distinctions in the populations labeled by each marker. CaMKIIα, a widely expressed intracellular kinase in excitatory neurons, is commonly used to target or label this neuronal population. In contrast, VGluT2, a presynaptic vesicular transporter in glutamatergic neurons, directly labels the presynaptic terminals and neurotransmitter phenotype of excitatory neurons. These two markers exhibit essential differences in their cellular localization and functional attributes. Collectively, these findings indicate that excitatory RN neurons, particularly those marked by CaMKII expression, modulate rotational angular velocity in a frequency-dependent manner without significantly altering postural configuration. Although VGluT2+ glutamatergic neurons are also involved in mediating rotational behavior, no significant frequency-dependent differences were observed within the tested range.

### 3.3. Excitatory Neurons in the RN Modulate Lateralized Movement Partly by Activating the Forelimbs

**Figure 4:**
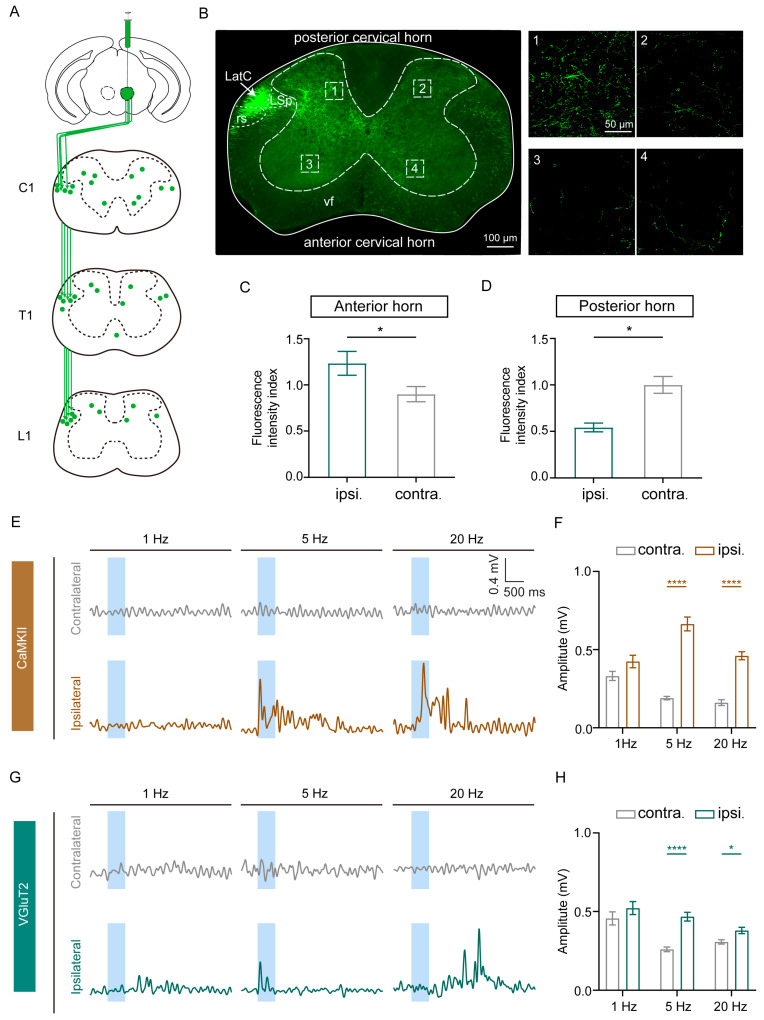
Excitatory neurons in the RN modulate lateralized movement partly by activating the forelimbs. (**A**) Schematic of RN excitatory neuron terminal distributions in the spinal cord. C—cervical, T—thoracic, L—lumbar. (**B**) Left panel: representative images of EYFP+ neuronal terminals in the cervical spinal cord. rs—rubrospinal tract, LatC—lateral cervical nucleus, LSp—lateral spinal nucleus. Scale bar, 100 μm. Right panel: magnifications of the right boxed areas showing RN excitatory neuron projections in the anterior and posterior horns on the ipsilateral and contralateral sides relative to the injected RN. Scale bar, 50 μm. (**C**,**D**) Comparison of the fluorescence density index between the ipsilateral and contralateral sides of the anterior horn (**C**) and the posterior horn (**D**); fluorescence density index = fluorescence density/mean fluorescence density of the contralateral side. Wilcoxon test, * *p* < 0.05. (**E**,**G**) Example EMG recording showing the activity of both the contralateral and ipsilateral forelimb muscles, and the increased activity ipsilaterally following photo-activation of RN CaMKII+ neurons (**E**) and VGluT2+ neurons (**G**) at 5 and 20 Hz for a duration of 500 ms. (**F**,**H**) Comparison of peak EMG signal amplitudes upon the optogenetic stimulation of red nucleus CaMKII+ neurons (**F**) and VGluT2+ neurons (**H**) at different frequencies. Wilcoxon test, * *p* < 0.05 and **** *p* < 0.0001.

Excitatory neurons in the RN project directly to the contralateral lateral funiculus of the spinal cord via the rubrospinal tract [3,35,36]. Consistent with previous studies, viral tracing revealed a dense distribution of axon terminals in the left lateral funiculus (Figure 4A). Notably, at the cervical spinal cord level (Figure 4B), we observed a significant ipsilateral dominance of RN projection terminals in the ventral horn, with the quantitative analysis showing higher fluorescence intensity ipsilaterally than contralaterally (*p* = 0.0286). In contrast, the dorsal horn exhibited the opposite pattern, displaying a higher contralateral fluorescence intensity (*p* = 0.0286) (Figure 4C,D). This spatial distribution carries important functional implications. The ventral horn, as the final common pathway for motor output, contains α-motoneurons that directly innervate ipsilateral skeletal muscles [37,38]. Consequently, the RN may directly regulate ipsilateral motor neuron pools through synaptic connections within the ipsilateral ventral horn, thereby facilitating lateralized motor control on the same side of the body.

To validate this mechanism, we examined the forelimb motor output using electromyography (EMG). Selective activation of CaMKII+ neurons induced contractions specifically in the ipsilateral biceps brachii during optical stimulation at 5 Hz and 20 Hz, but not at 1 Hz. Moreover, at the same light power (10 mW), the amplitude of EMG signals in the ipsilateral forelimb was significantly higher during 5 Hz and 20 Hz stimulation compared to the contralateral side (Figure 4E,F). A similar electromyographic response pattern was observed upon the optical stimulation of VGluT2+ neurons, although the response latency for 20 Hz stimulation was significantly prolonged relative to 5 Hz (Figure 4G,H). These EMG findings confirm that the RN may employ frequency-specific mechanisms to regulate ipsilateral forelimb movement.

### 3.4. The RN Primarily Projects to the MVN and ZI

**Figure 5:**
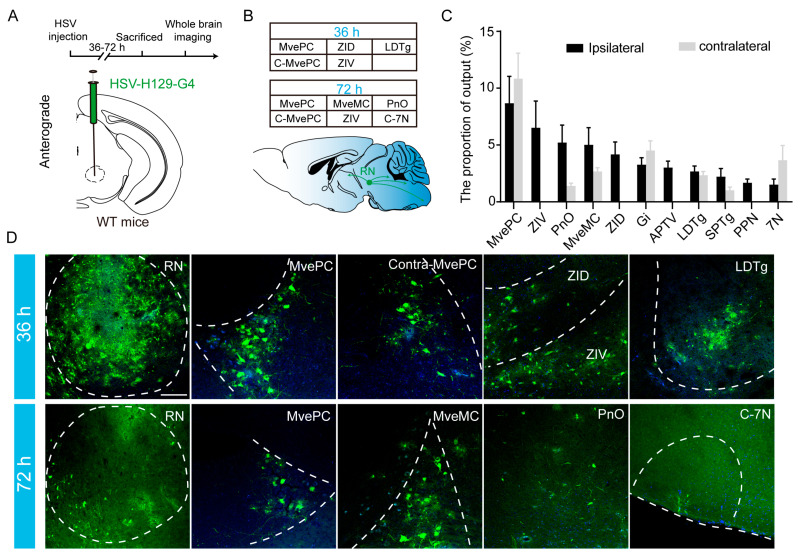
The RN primarily projects to the MVN and the ZI. (**A**) Top panel: the timeline of H129-G4 virus tracing of the right red nucleus output in wild-type mice. The brain was collected 36–72 h after the injection, and whole-brain imaging was performed on the slices. Bottom panel: schematic of the virus injection. (**B**) Top panel: the brain regions with the highest ranking for the RN outputs at 36 and 72 h post-injection. MvePC, medial vestibular nucleus, parvicellular part. MveMC, medial vestibular nucleus, magnocellular part. ZID, zona incerta, dorsal part. ZIV, zona incerta, ventral part. LDTg, laterodorsal tegmental nucleus. PnO, pontine reticular nucleus, oral part. 7N, facial nucleus. C, contralateral. Bottom panel: a simplified schematic of the red nucleus projection circuitry. (**C**) Statistical chart showing the proportions of various brain regions receiving the RN outputs, *n* = 6; the data are presented as means ± standard errors. SPTg, subpedencular tegmental nucleus. Gi, gigantocellular reticular nucleus. APTV, anterior pretectal nucleus, ventral part. PPN, pedunculopontine nucleus. Due to the variation in the infection time and individual differences, the brain regions downstream with output in at least half of the animals are shown in the figure. (**D**) Representative images of the RN injection site and RN-innervated downstream brain regions at 36 h and 72 h post-injection, with target neurons labeled with green fluorescence. Bar: 50 μm.

Using anterograde trans-synaptic tracing with H129-G4 [39], we systematically characterized the downstream output architecture of the RN at 36 and 72 h post-injection (Figure 5A). At the early time point (36 h), RN projections were primarily confined to the ipsilateral zona incerta dorsal part (ZID), ipsilateral zona incerta ventral part (ZIV), ipsilateral medial vestibular nucleus magnocellular part (MVeMC), and ipsilateral medial vestibular nucleus parvicellular part (MVePC), indicating a predominantly unilateral connectivity pattern. By 72 h post-injection, the projection network had expanded substantially, encompassing the original ipsilateral targets as well as new regions, including the contralateral MVePC, contralateral facial nucleus (7N), and the ipsilateral pontine reticular nucleus oral part (PnO) (Figure 5B,D). These findings suggest a temporally dynamic propagation of RN efferent pathways that are initially restricted to ipsilateral circuits before engaging broader bilateral motor-related structures. Quantitative analysis revealed that the bilateral medial vestibular nucleus (MVN), including both magnocellular and parvocellular subdivisions, as well as the ipsilateral zona incerta (ZI) and PnO, constitute the primary downstream targets of the RN. Additionally, the RN projects to the bilateral gigantocellular reticular nucleus (Gi), tegmental nuclei, 7N, and the ipsilateral pedunculopontine nucleus (PPN) (Figure 5C).

## 4. Discussion

This study demonstrates that excitatory neurons in the RN exhibit enhanced calcium signaling and increased firing rates during ipsilateral turning behaviors in T-maze experiments. Optogenetic activation of RN excitatory neurons elicited robust ipsilateral rotation with frequency-dependent increases in both the angular velocity and forelimb EMG amplitude. Neuroanatomical tracing confirmed RN projections to anterior horn of the spinal cord and revealed efferent connections to the ZI and MVN, establishing a neural substrate for lateralized motor regulation.

As a pivotal midbrain motor nucleus, the RN is known to regulate muscle tone, motor learning, conditioned motor responses, postural adjustments, jaw movements, and recovery after spinal injuries [18,40,41,42,43,44]. Previous studies have clearly demonstrated its pivotal role in regulating fine motor control of forelimb [19]. Notably, our recent work identified a contralateral movement preference during auditory-cued goal-directed tasks, wherein CaMKII+ RN activation induced contralateral bias modulated by ZI-RN inhibitory circuits [26]. Other studies have reported that RN neuronal encoding of movement direction correlates with velocity in cognitive tasks [45]. However, the current findings indicate that that the activation of RN excitatory neurons drives ipsilateral movement, suggesting state-dependent functional heterogeneity potentially mediated by distinct circuit priorities. Viral tracing identified the ZI as a key bidirectional target of the RN, coordinating motor adaptation across different behavioral states. Although direct evidence is currently lacking to demonstrate the regulation of directional movement specifically by the RN-ZI circuits, our previous research indicates that the inhibitory ZI-to-RN pathway is involved in regulating goal-directed movement [26]. The role of this reciprocal projection between the RN and ZI within the motor circuit remains to be further explored.

The brainstem serves as a critical hub for integrating supraspinal motor commands with spinal sensory feedback [46,47,48,49]. Midbrain locomotor regions (MLRs), including the PPN, precuneiform nucleus (pCnF), cuneiform nucleus (CnF), and mesencephalic reticular formation (mRT), regulate gait parameters and postural dynamics [46,47,50,51]. Turning behaviors involve specialized neural mechanisms, with distinct brainstem nuclei being activated during rapid and slow turns [52]. For instance, PPN D1 neurons promote gait while D2 neurons mediate ipsilateral turning, with partial colocalization of dopaminergic and glutamatergic populations [25]. Additionally, CHX10+ VGluT2+ Gi neurons project ipsilaterally to drive turning behaviors under basal ganglia control via PnO pathways [22,23,24]. Our study found that the activation of excitatory neurons in the RN can also induce ipsilateral turning behavior. Viral tracing revealed that the RN projects downstream to the MVN and Gi, with Gi neurons playing a critical role in locomotor gait programs and the MVN primarily coordinating head and body movements [53,54,55,56,57,58]. We postulate that the RN-MVN connection may contribute to coordinating vestibular and locomotor information relevant to navigation and orientation, although the precise nature of this potential functional coupling requires empirical validation. Additionally, sparse projections from the RN to the PPN were observed. These findings regarding the neural connections between the RN, MVN, Gi, PnO, and PPN provide further support for the role of the RN in modulating lateralized locomotor and postural control within the broader brainstem locomotor network.

In this study, we found that the density of RN neuronal projections to the ipsilateral anterior horn of the spinal cord was significantly higher than that to the contralateral side. As the final output site for motor commands, the spinal anterior horn directly controls skeletal muscle contraction through α-motor neurons, thereby governing limb movement. This anatomical observation was functionally validated by EMG recordings: optogenetic activation of excitatory neurons in the RN resulted in significantly higher EMG signal amplitudes in the ipsilateral forelimb compared to the contralateral side. These results are consistent with previous studies demonstrating that the contraction of the ipsilateral forelimb coupled with relaxation of the contralateral forelimb facilitates ipsilateral turning behavior. Notably, unlike Gi activation, which induces only transient EMG activity, RN stimulation elicited a more sustained effect. These findings suggest that the RN, through its predominant innervation of the ipsilateral spinal ventral horn, may serve as a key neural mechanism driving ipsilateral turning. The dominant topographic projection of RN excitatory neurons to ipsilateral motor neurons in the spinal ventral horn holds significant pathophysiological implications. In spinal cord injury (SCI) models, the RN is known to contribute to functional recovery [41], a mechanism that may partly rely on this preserved or reorganized ipsilateral descending pathway, which bypasses the damaged corticospinal tract and directly activates the ipsilateral spinal ventral motor neuron pool below the injury level, thereby driving movement in paralyzed limbs. Additionally, RN lesions can lead to ipsilateral limb tremors and coordination deficits [59,60,61], consistent with the ipsilateral spinal innervation pattern observed in this study. The direct regulation of α-motor neurons by the RN may explain why midbrain stroke patients often exhibit a combination of contralateral limb paralysis (due to corticospinal tract damage) and ipsilateral ataxia (due to rubrospinal tract injury). These insights provide a new perspective for understanding the underlying neural mechanisms of lateralized motor control and for developing targeted neuromodulatory strategies. Overall, this strict topographic organization enables the precise regulation of specific muscle groups, which is particularly critical for the recovery of fine motor functions.

One limitation of the current study is that although some studies have used CaMKIIα promoter-driven tools to target VGluT2+ neurons [62,63], these two markers fundamentally represent different neuronal populations. CaMKIIα is an intracellular kinase widely expressed in excitatory neurons [64], whereas VGluT2 is a synaptic vesicle transporter protein specific to glutamatergic neurons [65]. Since CaMKII+ and VGluT2+ neurons may only partially overlap, it remains necessary to perform double-labeling experiments to verify whether they indeed belong to the same neuronal subpopulation. Additionally, while HSV-H129 tracing can reflect the potential output pathways of the RN, identifying the distinct efferent projections of these functionally important neuronal subpopulations is crucial for a deeper understanding of the circuit organization and functional mechanisms of the RN. Furthermore, elucidating how the ZI contributes to RN-mediated turning will be a focus of our future research. The unclear anatomical boundary between the rostral magnocellular (RMC) and caudal parvicellular (RPC) subregions of the RN in mice also limits our ability to determine their distinct contributions to rotational behavior. Optogenetic functional MRI could provide valuable insights into whole-brain dynamics during RN activation, particularly RN–cerebellar interactions. Ongoing studies aim to identify key RN downstream nuclei involved in lateralized movement and to further elucidate the underlying mechanisms. Additionally, clarifying the differences in spinal projection patterns (ipsilateral vs. contralateral) and functions (e.g., fine motor control vs. gross turning) among distinct neurochemical subpopulations within the RN, as well as their dynamic changes under pathological conditions, will be central to understanding their pathophysiological significance.

## 5. Conclusions

Collectively, our results demonstrate that the RN serves as a specialized hub mediating ipsilateral motor output. These findings advance our understanding of asymmetric movement control within the central nervous system and may contribute to the development of targeted rehabilitation strategies for RN-related forelimb dysfunction. Clinically, this study provides experimental evidence for the role of the RN in motor dysfunction. While further validation of its translational potential is needed, these findings could inform future therapeutic interventions.

## Data Availability

All data are publicly available at https://data.mendeley.com/drafts/g27tsjxjh9 (accessed on 12 June 2025). Any additional information required to reanalyze the data reported in this paper is available from the lead contact upon request.

## Abbreviations

The following abbreviations are used in this manuscript:

RN	Red nucleus
ET	Essential tremor
PD	Parkinson’s disease
CaMKIIα	Calcium/calmodulin-dependent protein kinase II alpha
VGluT2	Vesicular glutamate transporter 2
PSTH	Peri-stimulus time histogram
AAV	Adeno-associated virus
ChR2	Channelrhodopsin-2
StGtACR2	Step-function inhibitory anion-conducting channelrhodopsin-2
EYFP	Enhanced yellow fluorescent protein
EMG	Electromyography
ZID	Zona incerta dorsal part
ZIV	Zona incerta ventral part
MVeMC	Medial vestibular nucleus magnocellular part
MVePC	Medial vestibular nucleus parvicellular part
7N	Facial nucleus (7th cranial nerve nucleus)
PnO	Pontine reticular nucleus oral part
MVN	Medial vestibular nucleus
ZI	Zona incerta
Gi	Gigantocellular reticular nucleus
PPN	Pedunculopontine nucleus
LDTg	Laterodorsal tegmental nucleus
SPTg	Subpeduncular tegmental nucleus
APTV	Anterior pretectal nucleus, ventral part
pCnF	Precuneiform nucleus
CnF	Cuneiform nucleus
RMC	Rostral magnocellular subregion (of the red nucleus)
RPC	Caudal parvicellular subregion (of the red nucleus)
PBS	Phosphate-buffered saline
PFA	Paraformaldehyde
BSA	Bovine serum albumin
LFP	Local field potential
HSV-1	Herpes simplex virus type 1
